# Shotgun Proteomic Analysis of Plasma from Dairy Cattle Suffering from Footrot: Characterization of Potential Disease-Associated Factors

**DOI:** 10.1371/journal.pone.0055973

**Published:** 2013-02-13

**Authors:** Dongbo Sun, Hong Zhang, Donghua Guo, Anguo Sun, Hongbin Wang

**Affiliations:** 1 College of Animal Science and Veterinary Medicine, Heilongjiang Bayi Agricultural University, Daqing High-tech Industrial Development Zone, Daqing, P. R. China; 2 Post-doctoral Mobile Station of Veterinary Medicine, Northeast Agricultural University, Harbin, P. R. China; 3 Research Center for Proteome Analysis, Institute of Biochemistry and Cell Biology, Shanghai Institutes for Biological Sciences, Chinese Academy of Sciences, Shanghai, P. R. China; University College Dublin, Ireland

## Abstract

The plasma proteome of healthy dairy cattle and those with footrot was investigated using a shotgun LC-MS/MS approach. In total, 648 proteins were identified in healthy plasma samples, of which 234 were non-redundant proteins and 123 were high-confidence proteins; 712 proteins were identified from footrot plasma samples, of which 272 were non-redundant proteins and 138 were high-confidence proteins. The high-confidence proteins showed significant differences between healthy and footrot plasma samples in molecular weight, isoelectric points and the Gene Ontology categories. 22 proteins were found that may differentiate between the two sets of plasma proteins, of which 16 potential differential expression (PDE) proteins from footrot plasma involved in immunoglobulins, innate immune recognition molecules, acute phase proteins, regulatory proteins, and cell adhesion and cytoskeletal proteins; 6 PDE proteins from healthy plasma involved in regulatory proteins, cytoskeletal proteins and coagulation factors. Of these PDE proteins, haptoglobin, SERPINA10 protein, afamin precursor, haptoglobin precursor, apolipoprotein D, predicted peptidoglycan recognition protein L (PGRP-L) and keratan sulfate proteoglycan (KS-PG) were suggested to be potential footrot-associated factors. The PDE proteins PGRP-L and KS-PG were highlighted as potential biomarkers of footrot in cattle. The resulting protein lists and potential differentially expressed proteins may provide valuable information to increase understanding of plasma protein profiles in cattle and to assist studies of footrot-associated factors.

## Introduction

Footrot is an acute and highly infectious disease of cattle that develops between the claws of the hoof and is caused by the Gram-negative anaerobic bacterium *Fusobacterium necrophorum*, which is present in the rumen and feces of normal cattle and their environment [1]–[3]. The disease is characterized by the presence of an interdigital lesion, swelling, moderate to severe lameness, and a separation of horny portions of the hoof from the sensitive tissues underneath. It has a serious impact on the production performance of diseased cattle, especially in dairy cattle. Since footrot was first reported by Adams in the Netherlands in 1960, many treatment and preventive measures had been developed for its control [4]–[6]. However, the disease is common in many cattle-raising countries, with incidence rates that vary from 10% to 25%.

Plasma is an amorphous and important component of blood and changes in the quantity and quality of plasma proteins are associated with physiological or pathological states in humans and other animals [7], [8]. Therefore plasma is an ongoing focus of research for elucidation of disease-associated factors [9]–[11]. The serum acute-phase protein haptoglobin has been reported to be a marker of inflammation in dairy cattle suffering from diseases of the hoof [12]. Additionally, the concentrations of serum sialic acids, inflammatory mediators and acute phase proteins have been proven to be significantly raised in lame cattle with interdigital dermatitis [13]. However, the plasma protein profiles of cattle with footrot are not fully understood, and there are still a great many unknown potential disease-associated proteins.

Proteomics techniques are an effective tool for characterization of protein profiles in plasma/serum samples and have been used widely to search for disease-associated factors and biomarkers [14]–[17]. Among current proteomics methods, the shotgun proteomics approach possesses the virtues of high efficiency, and time and labor savings, when compared with the two-dimensional electrophoresis (2-DE) combined with mass spectrometry (MS), and it is suitable for use as a high throughput technology for the identification of proteins in plasma or serum samples [18]–[22]. In this study, proteomic analysis of plasma proteins from dairy cattle with footrot and healthy cattle was performed using the shotgun proteomics approach based on liquid chromatography and tandem mass spectrometry (LC-MS/MS). Furthermore, we characterized the plasma protein profiles of healthy dairy cattle and those with footrot, and analyzed and verified potential footrot-associated factors or biomarkers. Our aim was to add basic information to increase understanding and the effective control of footrot in dairy cattle.

## Materials and Methods

### Ethics Statement

The animal experiments were approved by the Institutional Animal Care and Use Committee of Northeast Agricultural University, under the approved protocol number SRM-06.

### Preparation of Plasma Specimens

Eleven plasma specimens from diseased cattle were collected from a Holstein dairy herd that was suffering from an outbreak of footrot in the Daqing area of Heilongjiang Province, northeast China, in 2011. The blood samples (about 5 mL) from each cow were collected from the caudal vein into the evacuated blood collection tubes with anticoagulant according to the BD protocol. The diseased dairy cattle showed typical swelling of the skin between the claws of the hoof, which is one of the characteristic clinical signs of footrot; in hoof swabs of footrot-affected dairy cattle, the presence of the lktA gene of *F. necrophorum* was confirmed by PCR methods. After centrifugation at 3000×g for 8 min at 4°C, the resulting plasma specimens were centrifuged for a second time at 12000×g for 5 min at 4°C. Equal volumes of the 11 diseased plasma specimens were combined to form a pooled plasma sample, and 11 healthy plasma specimens from unaffected cattle in the affected dairy herd were pooled using the same procedure. After determination of the total protein concentration using Bradford’s method, according to the manufacturer’s instructions (Invitrogen, Carlsbad, CA), the two pooled plasma specimens, footrot and healthy, were stored at −80°C.

### SDS-PAGE Separation of Plasma Proteins

One hundred micrograms of protein from each plasma specimen was denatured at 100°C for 5 min in an equal volume of 2× protein loading buffer (0.1 M Tris buffer, pH 6.8, 4% SDS, 0.2% β-mercaptoethanol, 40% glycerol, and 0.002% bromophenol blue). The denatured plasma specimens were separated by 12.5% polyacrylamide gel electrophoresis (SDS-PAGE) in Tris-glycine-SDS buffer (10 mM Tris, 50 mM glycine, 0.1% SDS, pH 8.0) at 15 mA for 20 min and then 30 mA for 1.5 h in a mini-vertical electrophoresis system. The gels were then stained with Coomassie Brilliant Blue G250 (Invitrogen, Carlsbad, CA). The protein lane of each specimen was cut into four equal pieces.

### In-Gel Trypsin Digestion

The separated gel pieces for each specimen were destained with 30% ACN/100 mM NH_4_HCO_3_, and the destained gels were dried in a vacuum centrifuge. The in-gel proteins were reduced with dithiothreitol (10 mM DTT/100 mM NH_4_HCO_3_) for 30 min at 56°C, and subsequently alkylated with iodoacetamide (50 mM IAA/100 mM NH_4_HCO_3_) in the dark at room temperature for 30 min. The gel pieces were rinsed briefly with 100 mM NH_4_HCO_3_ and ACN, respectively. The gel pieces were digested overnight in 12.5 ng/mL trypsin in 25 mM NH_4_HCO_3_. The peptides were extracted three times with 60% ACN/0.1% TFA. The extracts were pooled and dried completely using a vacuum centrifuge.

### Liquid Chromatography and Tandem Mass Spectrometry (LC−MS/MS)

The EttanTM MDLC system (GE Healthcare) was used for desalting and separation of the tryptic peptide mixtures. In this system, samples were desalted on RP trap columns (Zorbax 300 SB C18, Agilent Technologies), and separated on a RP column (150 µm i.d., 100 mm length, Column technology Inc., Fremont, CA). Mobile phase A (0.1% formic acid in HPLC-grade water) and mobile phase B (0.1% formic acid in acetonitrile) were selected. Subsequently, 20 µg of each tryptic peptide mixture was loaded onto the column, and separation was performed at a flow rate of 2 µL/min using a linear gradient of 4–50% B for 60 min. An LTQ Velos (Finnigan, San Jose, CA), equipped with an electrospray interface, was connected to the LC setup for detection of the eluted peptides. Data-dependent MS/MS spectra were obtained simultaneously. Each scan cycle consisted of one full MS scan in profile mode followed by 20 MS/MS scans in centroid mode, with the following Dynamic ExclusionTM settings: repeat count 2, repeat duration 30 s, exclusion duration 90 s.

### Protein Identification

The acquired MS/MS spectra were searched automatically against the protein database for Bovidae proteins in NCBI using the BioworksBrowser rev. 3.1 (Thermo Electron, San Jose, CA). The protein identification results were extracted from SEQUEST outfiles with BuildSummary which combined the peptide sequences into proteins and deleted redundant proteins [23]. The peptides were constrained to be tryptic, and up to two missed cleavages were allowed. Carbamidomethylation of cysteines was treated as a fixed modification, whereas oxidation of methionine residues was considered as a variable modification. The mass tolerance allowed for the precursor ions was 2.0 Da and that for the fragment ions was 0.8 Da. The protein identification criteria were based on Delta CN (≥0.1) and cross-correlation scores (Xcorr, one charge ≥1.9, two charges ≥2.2, three charges ≥3.75). The high-confidence proteins were determined by the standard of unique peptide count>or = 2 or a unique peptide count = 1 but total count>or = 4. Gene Ontology (GO) categories of the high-confidence proteins were performed with the DAVID web tool available at the website http://david.abcc.ncifcrf.gov/according to the protein geninfo identifier (GI) accession numbers [24], [25].

### ELISA

The concentrations of immunoglobulin G (IgG) in the plasma samples of both healthy dairy cattle and those affected by footrot were detected by Bovine IgG ELISA kit (Xinyue Biotechnology Co., Ltd., Shanghai, China) according to the manufacturer’s instructions, respectively. Briefly, 40 µL of the plasma samples (1∶50 dilution in PBS) of both healthy and footrot-affected dairy cattle was added to wells of ELISA plate coated by monoclonal antibody (McAb) against Bovine IgGs, respectively, and then 10 µL of biotin-labeled McAb against Bocine IgGs and 50 µL of streptavidin-HRP conjugates were added to the wells of ELISA plate, respectively. After incubation at 37°C for 1 h, the ELISA plate was washed three times using PBST (0.5% (v/v) Tween-20, PBS, pH 7.4). Color development was carried out using TMB solution as the substrate, and the reaction was stopped with 50 µL of 2 M H_2_SO_4_. The absorbance at 450 nm was measured. In ELISA, Bovine IgGs standard (320 µg/mL, 160 µg/mL, 80 µg/mL, 40 µg/mL, and 20 µg/mL) was used to prepare a standard curve according to the ELISA procedure described above. IgG concentrations of the plasma samples from healthy dairy cattle and those affected by footrot were calculated according to the standard curve of Bovine IgGs standard. Data were analyzed using a two-tailed, paired Student’s t test in the Microsoft Excel 2007 Windows software.

## Results

### Identification of Plasma Proteins

**Figure 1:**
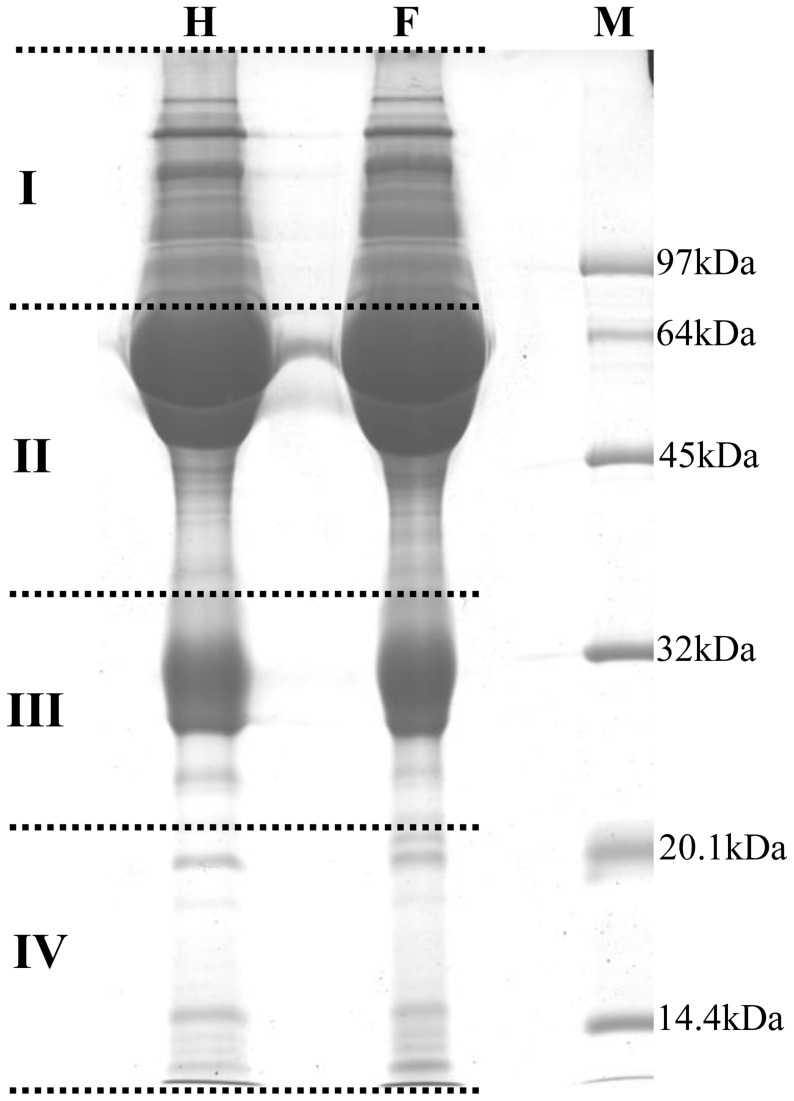
Separation of plasma proteins by SDS-PAGE. Lane M, Protein Marker (14.4 kDa–97 kDa); Lane F, the 11 pooled plasma proteins from footrot-affected dairy cattle; Lane H, the 11 pooled healthy plasma proteins from unaffected dairy cattle.

The plasma proteins of healthy dairy cattle and those with footrot were separated by SDS-PAGE, respectively, and each separated gel was cut into four pieces that were equal in size (Fig. 1). After in-gel trypsin digestion, the plasma proteins from healthy and footrot-affected dairy cattle were analyzed using the shotgun LC-MS/MS proteomics technique. A total of 648 proteins and 712 proteins were identified from plasma samples of healthy dairy cattle and those with footrot, respectively (Table 1). Of the 648 proteins from healthy dairy cattle, the numbers of non-redundant proteins and high-confidence proteins were 234 (36.11%) and 123 (18.98%), respectively. Of the 712 proteins from footrot-affected dairy cattle, the numbers of non-redundant proteins and high-confidence proteins were 272 (38.20%) and 138 (19.38%), respectively. Between the two plasma specimens, a total of 22 potential differentially expressed proteins were found, of which 16 proteins (2.24%) occurred in footrot plasma and 6 proteins (0.92%) were present in healthy plasma.

**Table 1:** Numbers of the proteins identified from healthy and footrot plasma samples.

	Healthy plasma	Footrot plasma
Total proteins no.	648 (100%)	712 (100%)
Non-redundant proteins no.	234 (36.11%)	272 (38.20%)
High-confidence proteins no.	123 (18.98%)	138 (19.38%)
Differential proteins no.	6 (0.92%)	16 (2.24%)

### Characterization of Plasma Protein Profile

**Figure 2:**
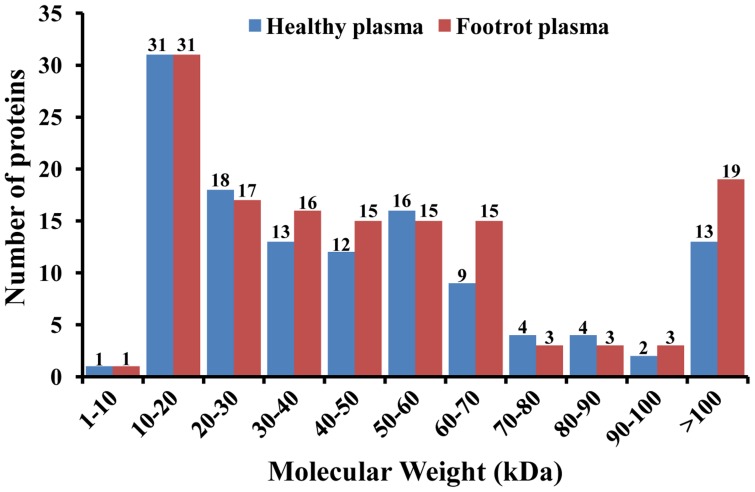
Distributions of molecular weight of the high-confidence proteins from healthy plasma and footrot plasma.

**Figure 3:**
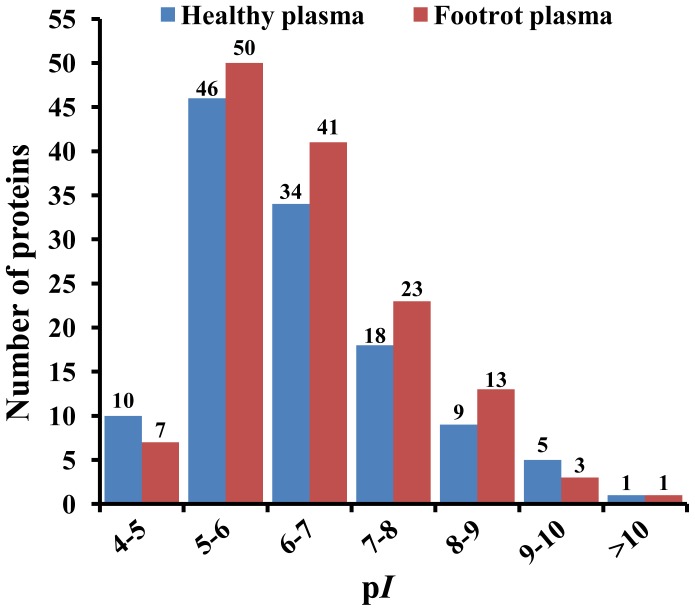
Distributions of isoelectric point (p*I*) of the high-confidence proteins from healthy plasma and footrot plasma.

The lists of the high-confidence proteins from healthy and footrot plasma samples are shown in Table 2 and Table 3, respectively. The molecular weight analysis of healthy and footrot plasma samples is shown in Fig. 2. In healthy plasma, the molecular weight of the high-confidence proteins ranged between 6.33 kDa and 249.56 kDa; proteins from 10 kDa to 70 kDa accounted for 80.49% (99/123), and proteins of greater than 100 kDa accounted for 10.57% (13/123). In footrot plasma, the molecular weight of the high-confidence proteins ranged between 4.31 kDa and 353.34 kDa; proteins from 10 kDa to 70 kDa accounted for 78.98% (109/138), and proteins of greater than 100 kDa accounted for 13.77% (19/138). Between the plasma proteins of healthy and footrot-affected cattle, there were significant differences in the molecular weight distributions at 30 kDa–50 kDa, 60 kDa–70 kDa and >100 kDa. Analysis of the isoelectric points (p*I*) of healthy and footrot plasma samples is shown in Fig. 3. In healthy plasma, the p*I* of the high-confidence proteins ranged between 4.31 and 10.71, and proteins from p*I* 5 to p*I* 9 accounted for 86.99% (107/123). In footrot plasma, the p*I* of the high-confidence proteins ranged between 4.31 and 10.71, and proteins from p*I* 5 to p*I* 9 accounted for 92.03% (127/138). Between healthy and footrot plasma proteins, there was a significant difference in the p*I* distribution at p*I* 4–10.

**Table 2:** The high-confidence proteins in the plasma sample of healthy dairy cattle.

No.	Protein name	Accession no.	No.	Protein name	Accession no.	No.	Protein name	Accession no.	No.	Protein name	Accession no.
1	chain B, structure of mammalian C3 with an intact thioester at 3a resolution	gi|110590847|	32	Ig gamma-2 chain C region	gi|89611|	63	alpha-1B-glycoprotein precursor	gi|114053019|	94	Ig L chain VJ region	gi|4680177|
2	alpha-2-macroglobulin precursor	gi|157954061|	33	IGK protein	gi|154425814|	64	Ig L chain V region	gi|2323404|	95	C-reactive protein precursor	gi|76611918|
3	serum albumin precursor	gi|30794280|	34	transthyretin	gi|3915182|	65	alpha macroglobulin	gi|226283|	96	kininogen 1	gi|28461175|
4	unnamed protein	gi|110292444|	35	ceruloplasmin isoform 1	gi|119884990|	66	Ig gamma-1 chain	gi|346578|	97	Ig L chain V region	gi|2555153|
5	apolipoprotein A-I	gi|113988|	36	prothrombin	gi|135806|	67	Ig L chain V region	gi|2323384|	98	Ig L chain V region	gi|5802440|
6	fibronectin	gi|462100|	37	predicted: similar to MGC127066 protein	gi|119909599|	68	Ig L chain V region	gi|2323390|	99	apoN protein	gi|86438511|
7	transferrin	gi|113911795|	38	chain B, carbonmonoxy liganded bovine hemoglobin Ph 5.0	gi|12084213|	69	serum amyloid A-4 protein	gi|122138722|	100	SERPIND1 protein	gi|151556981|
8	serum albumin precursor	gi|57164373|	39	complement factor H	gi|115298718|	70	complement component C4	gi|1229|	101	SHBG protein	gi|148743950|
9	similar to complement component 4A	gi|119915491|	40	plasminogen precursor	gi|27806815|	71	predicted: serpin A3-3 isoform 3	gi|119914040|	102	anti-respiratory syncytial virus Ig lambda chain V region	gi|508836|
10	complement C4 precursor	gi|119915494|	41	histidine-rich glycoprotein precursor	gi|27806875|	72	Ig mu H chain V region	gi|98991290|	103	Ig L chain V region	gi|2555151|
11	apolipoprotein A-IV	gi|118598012|	42	coagulation factor II	gi|75948172|	73	protein HP-25 homolog 1 precursor	gi|114050753|	104	IgG3 H chain C region	gi|1575495|
12	IGHM protein	gi|151554795|	43	hemoglobin subunit beta-A	gi|122539|	74	conglutinin	gi|461774|	105	predicted: C4b-binding protein alpha chain-like	gi|119922208|
13	inter-alpha-trypsin inhibitor heavy chain H1	gi|122142424|	44	leucine-rich alpha-2-glycoprotein precursor	gi|114051379|	75	lipocalin/cytosolic fatty-acid binding protein	gi|157831280|	106	tetranectin	gi|108861909|
14	ITIH2 protein	gi|146186952|	45	vitronectin precursor	gi|78045497|	76	Ig variable region	gi|2353754|	107	CD5 antigen-like precursor	gi|156120885|
15	immunoglobulin gamma 1 heavy chain constant region	gi|91982959|	46	predicted: similar to endopin 2B	gi|119914034|	77	Ig γ chain V region	gi|94961524|	108	alpha-2-HS-glycoprotein	gi|154426172|
16	complement factor B	gi|146345391|	47	alpha-1-acid glycoprotein	gi|121957959|	78	alpha-1-antiproteinase precursor	gi|27806941|	109	KRT4 protein	gi|134024768|
17	Vl1a protein	gi|86438072|	48	chain C, carbonmonoxy liganded bovine hemoglobin Ph 7.2	gi|12084218|	79	angiotensinogen	gi|74354323|	110	C1QC protein	gi|92097580|
18	anti-testosterone antibody	gi|440|	49	vitamin D-binding protein	gi|85701291|	80	Ig mu H chain V region	gi|98991272|	111	extracellular matrix protein 1 precursor	gi|153791660|
19	IGHG1 protein	gi|151554383|	50	complement factor I precursor	gi|84000165|	81	apolipoprotein E precursor	gi|27806739|	112	cylicin-2	gi|2498277|
20	ITI heavy chain H4	gi|122140331|	51	predicted: complement component 5	gi|119901003|	82	Ig heavy chain	gi|1322326|	113	complement C1s subcomponent	gi|146286057|
21	hypothetical protein LOC505478	gi|115497996|	52	Ig mu chain - sheep	gi|478694|	83	Ig H chain V region	gi|3834645|	114	predicted: hypothetical protein isoform 8	gi|119912360|
22	IGL@ protein	gi|74353860|	53	IgG L chain V region	gi|2323402|	84	complement component C8 gamma chain precursor	gi|119904354|	115	fibrinogen alpha chain precursor	gi|75812954|
23	MGC127066 protein	gi|148744106|	54	IgG L chain V region	gi|2323392|	85	Ig H chain V region	gi|24459869|	116	factor XIIa inhibitor	gi|1706733|
24	unknown protein	gi|148878143|	55	pigment epithelium-derived factor precursor	gi|27806487|	86	Ig mu H chain V region	gi|98991286|	117	Ig mu H chain V region	gi|98991296|
25	IgG1 H chain constant region	gi|7547266|	56	AMBP protein precursor	gi|2506821|	87	hemoglobin subunit alpha	gi|110831923|	118	Ig lambda L chain V region	gi|1276619|
26	chain B, crystal structure of bovine plasma copper-containing amine oxidase	gi|61680008|	57	histidine-rich glycoprotein	gi|1072452|	88	hemoglobin subunit beta-A	gi|122540|	119	angiotensinogen	gi|1703309|
27	gelsolin	gi|122140093|	58	paraoxonase 1	gi|86826758|	89	coagulation factor V	gi|2493324|	120	predicted: similar to mannosidase	gi|119888855|
28	predicted:similar to alpha-2-macroglobulin precursor	gi|119893044|	59	hibernation-associated plasma protein HP-20-like	gi|86438473|	90	complement component C7	gi|126361384|	121	Ig lambda-6a L chain V region	gi|2746695|
29	IgG lambda light chain	gi|15088675|	60	histidine-rich glycoprotein = factor XIIIa substrate	gi|1683228|	91	ADIPOQ protein	gi|146186777|	122	Ig lambda L chain V region	gi|1276629|
30	IgM H chain constant region	gi|28592070|	61	endopin-1 precursor	gi|75068363|	92	antithrombin-III precursor	gi|109940161|	123	Ig lambda L chain V region	gi|975858|
31	IgG H chain constant region	gi|3834667|	62	serpin A3-3 precursor	gi|84000377|	93	protein HP-25 homolog 2 precursor	gi|114052108|			

**Table 3:** The high-confidence proteins in the plasma sample of footrot dairy cattle.

No.	Protein name	Accession no.	No.	Protein name	Accession no.	No.	Protein name	Accession no.	No.	Protein name	Accession no.
1	chain B, structure of mammalian C3 with an intact thioester at 3a resolution	gi|110590847|	36	Ig lambda light chain	gi|15088675|	71	predicted:similar to H factor 1	gi|119922668|	106	Ig light chain variable region	gi|2555151|
2	alpha-2-macroglobulin precursor	gi|157954061|	37	IgM heavy chain constant region	gi|28592070|	72	hemoglobin subunit beta-A	gi|122540|	107	apolipoprotein A-II	gi|109940051|
3	transferrin	gi|113911795|	38	Ig _γ_ 1 heavy chain constant region	gi|91982959|	73	predicted: mammalian C3	gi|119894726|	108	Ig mu heavy chain variable region	gi|98991290|
4	serum albumin precursor	gi|30794280|	39	predicted: similar to MGC127066 protein	gi|119909599|	74	clusterin	gi|151555910|	109	hemoglobin subunit alpha	gi|110831923|
5	unnamed protein product	gi|110292444|	40	bovine carbonmonoxy Hb	gi|14488451|	75	Ig light chain variable region	gi|2323392|	110	apolipoprotein E precursor	gi|27806739|
6	predicted: similar to complement component 4A	gi|119915491|	41	IgG1 heavy chain constant region	gi|7547266|	76	Ig light chain variable region	gi|2323402|	111	extracellular matrix protein 1 precursor	gi|153791660|
7	albumin	gi|229552|	42	predicted:similar to alpha-2-macroglobulin precursor	gi|119893044|	77	Ig λ chain C region - sheep	gi|109030|	112	haptoglobin precursor	gi|2144490|
8	complement C4 precursor	gi|119915494|	43	leucine-rich alpha-2-glycoprotein precursor	gi|114051379|	78	Ig _γ_-1 chain - sheep	gi|346578|	113	predicted:similar to H factor 1	gi|119925635|
9	apolipoprotein A-I precursor	gi|113988|	44	IgG2a heavy chain constant region	gi|1699167|	79	Ig light chain variable region	gi|2323398|	114	complement factor I precursor	gi|84000165|
10	ITI heavy chain H4	gi|122140331|	45	predicted:C4b-binding protein alpha chain	gi|76677514|	80	protein AMBP	gi|2506821|	115	conglutinin precursor	gi|461774|
11	inter-alpha (globulin) inhibitor H4	gi|59857769|	46	serpin A3-3 precursor	gi|84000377|	81	protein HP-25 homolog 1 precursor	gi|114050753|	116	alpha-2-antiplasmin precursor	gi|1168249|
12	chain B, crystal structure of bovine plasma copper-containing amine oxidase	gi|61680008|	47	complement C6 precursor	gi|118601082|	82	factor XIIa inhibitor	gi|1706733|	117	SHBG protein	gi|148743950|
13	serum albumin precursor	gi|113582|	48	fibronectin (FN)	gi|462100|	83	alpha-1-B glycoprotein	gi|114053019|	118	complement component C7	gi|126361384|
14	haptoglobin	gi|94966763|	49	Ig gamma-2 chain C region	gi|89611|	84	Ig light chain variable region	gi|2555153|	119	Ig lambda light chain V region	gi|1276619|
15	IGHM protein	gi|151554795|	50	alpha-1acid glycoprotein precursor	gi|121957959|	85	vitamin D-binding protein	gi|85701291|	120	Ig lambda light chain V region	gi|975854|
16	predicted: ceruloplasmin isoform 1	gi|119884990|	51	SERPIND1 protein	gi|151556981|	86	chain C, carbonmonoxy Hb	gi|14488450|	121	CD5 antigen-like precursor	gi|156120885|
17	gelsolin	gi|122140093|	52	paraoxonase 1	gi|86826758|	87	complement component C4	gi|1229|	122	centromere protein F	gi|119908705|
18	Complement factor H	gi|115298718|	53	vitronectin	gi|78045497|	88	kininogen-2	gi|125506|	123	desmoplakin	gi|119915951|
19	Ig M heavy chain secretory form	gi|24496448|	54	C4b-binding protein alpha chain	gi|2493791|	89	uncharacterized protein LOC790886 precursor	gi|157279963|	124	predicted:KIAA0683 gene product-like	gi|119935194|
20	Ig mu heavy chain constant region	gi|33413902|	55	pigment epithelium-derived factor precursor	gi|27806487|	90	Ig light chain variable region	gi|2323390|	125	coagulation factor V precursor	gi|2493324|
21	complement factor B	gi|146345391|	56	predicted:similar to endopin 2B	gi|119914034|	91	SERPINA3-8	gi|151554250|	126	Ig lambda light chain V region	gi|1276609|
22	apolipoprotein A-IV precursor	gi|118598012|	57	transthyretin	gi|3915182|	92	amyloid A4 protein precursor	gi|122138722|	127	phosphatidylinositol-glycan-specific phospholipase D precursor	gi|27807363|
23	ITI heavy chain H1	gi|122142424|	58	serpin A3-5 precursor	gi|126165236|	93	IgG3 heavy chain C region	gi|1575495|	128	predicted:similar to superficial zone protein	gi|119908681|
24	ITIH2 protein	gi|146186952|	59	hemopexin precursor	gi|77736171|	94	hemoglobin subunit beta	gi|122654|	129	predicted peptidoglycan protein L	gi|119894607|
25	Vl1a protein	gi|86438072|	60	histidine-rich glycoprotein precursor	gi|27806875|	95	alpha-2-HS-glycoprotein	gi|154426172|	130	Ig lambda light chain V region	gi|975858|
26	hypothetical protein LOC505478	gi|115497996|	61	hibernation-associated plasma protein HP-20-like	gi|86438473|	96	complement C5a anaphylatoxin precursor	gi|119901003|	131	chain A, plasma retinol-binding protein	gi|157831280|
27	IGL@ protein	gi|74353860|	62	predicted:serpin A3-3 isoform 3	gi|119914040|	97	SERPINA10 protein	gi|148745555|	132	Ig λ-6a light chain variable region	gi|2746695|
28	Ig heavy chain constant region	gi|3834667|	63	Ig mu chain - sheep	gi|478694|	98	afamin precursor	gi|76656723|	133	Ig λ chain precursor V region	gi|109032|
29	IGK protein	gi|115545495|	64	cleaved bovine antithrombin Iii	gi|157838186|	99	inhibitor of carbonic anhydrase precursor	gi|114053269|	134	ovarian and testicular apolipoprotein N precursor	gi|51491835|
30	SERPINA3 protein	gi|86438018|	65	protein HP-25 homolog 2 precursor	gi|114052108|	100	Ig light chain variable region	gi|2323378|	135	apolipoprotein C-III	gi|78099960|
31	plasminogen	gi|27806815|	66	Ig light chain variable region	gi|2323404|	101	Ig lambda chain V region	gi|508836|	136	apolipoprotein D	gi|122142930|
32	CCP modules 3-12	gi|1419424|	67	prothrombin precursor	gi|27806947|	102	Ig light chain VJ region	gi|4680177|	137	keratan sulfate proteoglycan	gi|1708876|
33	IGL@ protein	gi|148744106|	68	histidine-rich glycoprotein	gi|1072452|	103	Ig light chain variable region	gi|2323384|	138	predicted:hypothetical protein	gi|119903609|
34	unknown protein	gi|151556360|	69	C8 gamma chain precursor	gi|119904354|	104	Ig variable region	gi|2353754|			
35	anti-testosterone antibody	gi|440|	70	tetranectin	gi|108861909|	105	C-reactive protein precursor	gi|76611918|			

**Figure 4:**
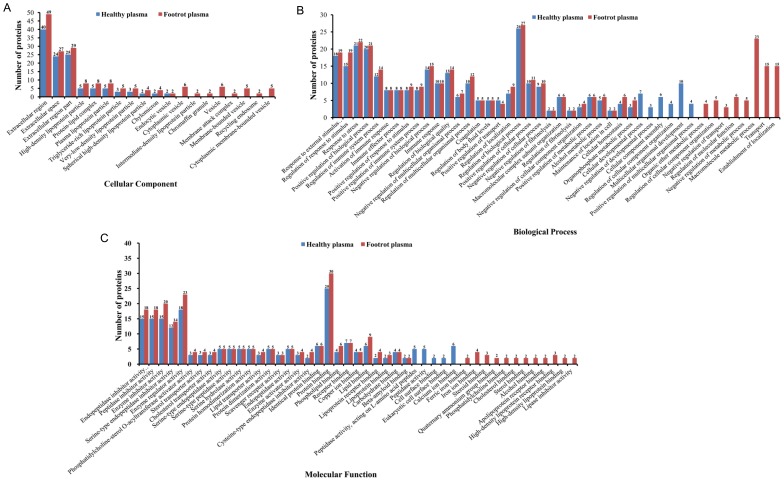
GO categories of the high-confidence proteins from healthy plasma and footrot plasma. A, Cellular component GO categories; B, Biological process GO categories; C, Molecular function GO categories.

To investigate the function of the high-confidence proteins we had identified further, the GO categories were ascertained to characterize them according to cellular components, biological processes and molecular functions. The cellular component categories are shown in Fig. 4A. In the healthy plasma, 96 proteins of the 123 high-confidence proteins were annotated and categorized in 11 groups of cellular components; In the footrot plasma, 107 proteins of the 138 high-confidence proteins were annotated and categorized in 19 groups of cellular components. The common rich cellular components for both healthy and footrot plasma were focused in the extracellular region (GO:0005576), extracellular space (GO:0005615) and extracellular region parts (GO:0044421), respectively. Compared with the healthy plasma sample, eight differential cellular components, cytoplasmic vesicle (GO:0031410), intermediate-density lipoprotein particle (GO:0034363), chromaffin granule (GO:0042583), vesicle (GO:0031982), membrane attack complex (GO:0005579), membrane-bound vesicle (GO:0031988), recycling endosome (GO:0055037), and cytoplasmic membrane-bounded vesicle (GO:0016023), were found in the footrot plasma sample.

The biological process categories are shown in Fig. 4B. Ninety proteins out of the 123 high-confidence proteins in the healthy plasma were found in 36 groups of biological processes, and 101 proteins of the 138 high-confidence proteins of the footrot plasma were categorized in 38 groups of biological processes. Thirty common biological processes were presented in both healthy and footrot plasma samples, among which regulation of biological process (GO:0050789), response to stress (GO:0006950), positive regulation of biological process (GO:0048518), response to external stimulus (GO:0009605), regulation of response to stimulus (GO:0048583), negative regulation of biological process (GO:0048519), regulation of biological quality (GO:0065008) and regulation of immune system process (GO:0002682) were common rich biological processes, respectively. Fourteen differential biological processes were found, of which six occurred in healthy plasma and eight in footrot plasma. In footrot plasma, the eight differential biological processes were involved in organic ether metabolic process (GO:0018904), regulation of cellular component organization (GO:0051128), negative regulation of transport (GO:0051051), regulation of molecular function (GO:0065009), negative regulation of metabolic process (GO:0009892), macromolecule metabolic process (GO:0043170), transport (GO:0006810) and establishment of localization (GO:0051234), of which macromolecule metabolic process was a rich biological process.

The result for molecular function is shown in Fig. 4C. Ninety-six proteins out of the 123 high-confidence proteins in the healthy plasma were involved in 33 groups of molecular functions, and 106 proteins of the 138 high-confidence proteins in the footrot plasma were involved in 40 groups of molecular functions. Between the healthy and footrot plasma samples, there were 28 identical molecular function categories, of which protein binding (GO:0005515), enzyme regulator activity (GO:0030234), endopeptidase inhibitor activity (GO:0004866), peptidase inhibitor activity (GO:0030414) and enzyme inhibitor activity (GO:0004857) were common rich molecular functions, respectively. A total of 17 differential molecular functions were found, of which five occurred in healthy plasma and 12 in footrot plasma. In the footrot plasma, 11 of the 12 differential molecular functions focused on binding activity, including ferric iron binding (GO:0008199), iron ion binding (GO:0005506), steroid binding (GO:0005496), cholesterol binding (GO:0015485), apolipoprotein receptor binding (GO:0034190), alcohol binding (GO:0043178), quaternary ammonium group binding (GO:0050997), phosphatidylcholine binding (GO:0031210), sterol binding (GO:0032934), high-density lipoprotein receptor binding (GO:0070653) and high-density lipoprotein binding (GO:0008035). In the healthy plasma, the five differential molecular functions were involved in calcium ion binding (GO:0005509), eukaryotic cell surface binding (GO:0043499), cell surface binding (GO:0043498), peptidase activity (GO:0008233), and peptidase activity acting on L-amino acid peptides (GO:0070011).

### Analysis of Potential Footrot-associated Proteins

**Figure 5:**
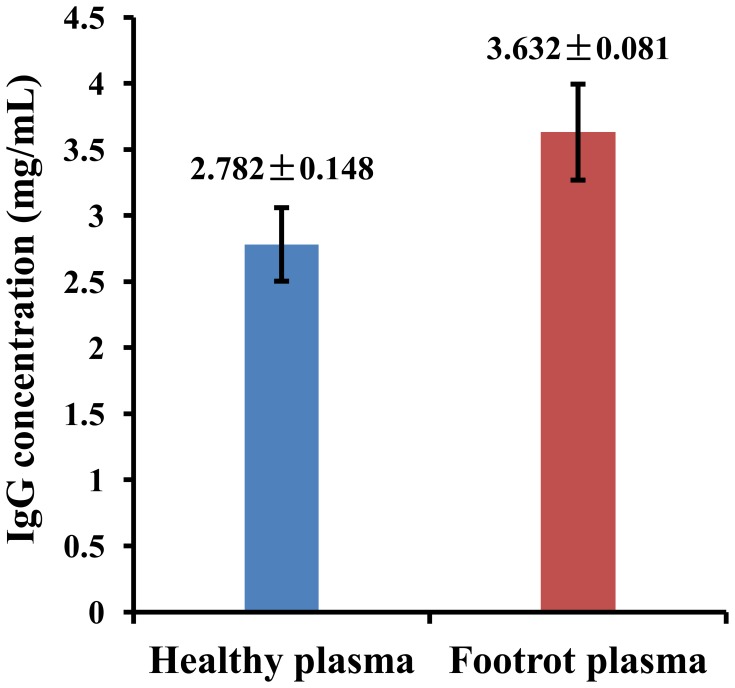
ELISA of the IgGs in the plasma samples of both healthy dairy cattle and those affected by footrot. *Note*. all data are expressed as mean ± SD, p = 0.0027 (*p*<0.01).

The proteins that showed potential differential expression between healthy and footrot plasma samples were searched for among the sets of high-confidence proteins using the geninfo identifier (GI) number of each protein, and the lists of potential differential expression (PDE) proteins are shown in Table 4. Six PDE proteins were found in plasma samples from healthy dairy cattle, involving in regulatory proteins (ADIPOQ protein and angiotensinogen), cytoskeletal proteins (KRT4 protein and cylicin-2), and coagulation factor (fibrinogen alpha chain precursor). Of six PDE proteins, ADIPOQ protein, fibrinogen alpha chain precursor and angiotensinogen could be associated with the pathogenesis of footrot in dairy cattle. 16 PDE proteins were found in plasma samples from dairy cattle with footrot, involving in immunoglobulins (IgGs), innate immune recognition molecules (predicted peptidoglycan recognition protein L), acute phase proteins (haptoglobin, haptoglobin precursor, afamin precursor), regulatory proteins (SERPINA10 protein, mammalian C3, alpha-2-antiplasmin precursor and apolipoprotein-D), and cell adhesion and cytoskeletal proteins (keratan sulfate proteoglycan, centromere protein F, desmoplakin, similar to superficial zone protein). Of 16 PDE proteins, haptoglobin, SERPINA10 protein, afamin precursor, haptoglobin precursor, predicted peptidoglycan recognition protein L (PGRP-L), apolipoprotein D, and keratan sulfate proteoglycan (KS-PG) were suggested to be disease-associated proteins or biomarkers according to current research reports. Furthermore, ELISA result indicated that the IgG concentration of healthy and footrot plasma samples was 2.782±0.148 mg/mL and 3.632±0.081 mmol/L, respectively. The IgG concentration in footrot plasma sample was significantly higher than those of healthy plasma sample (*p*<0.01) (Fig. 5).

**Table 4:** The potential differential proteins between healthy and footrot plasma samples.

The differential proteins in footrot plasma sample							
No.	Accession no.	Protein name	MW (Da)	p*I*	Peptides no.	Unique peptides no.	Cover percent
1	gi|94966763|	haptoglobin	44859.08	7.83	36	17	51.12%
2	gi|119894726|	predicted:mammalian C3	180556.4	6.46	7	4	3.47%
3	gi|109030|	Ig λ chain C region - sheep	11311.56	8.46	20	3	33.33%
4	gi|157279963|	uncharacterized protein LOC790886 precursor	45428.42	7.72	9	3	8.61%
5	gi|148745555|	SERPINA10 protein	51988.26	6.05	4	3	8.19%
6	gi|76656723|	afamin precursor	69562.06	5.63	4	3	7.45%
7	gi|2144490|	haptoglobin precursor	4680.19	4.64	4	2	48.89%
8	gi|1168249|	alpha-2-antiplasmin precursor	54711	5.45	3	2	8.94%
9	gi|119908705|	centromere protein F	353344.7	5.01	2	2	1.17%
10	gi|119915951|	desmoplakin	332383.2	6.47	2	2	0.62%
11	gi|119935194|	predicted:KIAA0683 gene product-like	60081.36	5.3	2	2	6.02%
12	gi|1276609|	Ig lambda light chain V region	13510.09	7.7	2	2	16.03%
13	gi|119908681|	predicted:similar to superficial zone protein	133388.3	9.21	2	2	2.67%
14	gi|119894607|	predicted peptidoglycan recognition protein L	63486.22	6.47	2	2	7.11%
15	gi|122142930|	apolipoprotein D	21401.59	4.8	4	1	6.35%
16	gi|1708876|	keratan sulfate proteoglycan	38756.48	5.93	4	1	5.26%
**The differential proteins in healthy plasma sample**							
**No.**	**Accession no.**	**Protein name**	**MW (Da)**	**p** ***I***	**Peptides no.**	**Unique peptides no.**	**Cover percent**
1	gi|146186777|	ADIPOQ protein	26133.2	5.46	3	3	17.50%
2	gi|134024768|	KRT4 protein	58046.33	7.47	3	2	3.83%
3	gi|2498277|	cylicin-2	53561.72	9.76	2	2	3.89%
4	gi|119912360|	predicted: hypothetical protein isoform 8	86933.48	4.81	2	2	2.84%
5	gi|75812954|	fibrinogen alpha chain precursor	67012.11	6.73	2	2	4.72
6	gi|1703309|	angiotensinogen	51304.09	6.54	4	1	2.73%

## Discussion

In our study, the shotgun proteomics technique was used to identify the plasma protein profiles of dairy cattle in 11 pooled healthy specimens and 11 pooled footrot specimens, respectively. A total of 648 proteins and 712 proteins were identified from the plasma samples of healthy dairy cattle and dairy cattle affected by footrot, respectively, according to the stringent filtering parameters of Delta CN (≥0.1) and Xcorr (one charge ≥1.9, two charges ≥2.2, three charges ≥3.75). The total number of proteins identified (648 or 712) was significantly higher than the number identified in human plasma (622) by the shotgun proteomics technique [21]. Although the highly abundant plasma proteins, such as albumin, IgG, and IgA, were not removed in our experiment, we still obtained highly enriched protein samples from the plasma samples of dairy cattle. This result suggests that the presence of the highly abundant plasma proteins has little effect on the identification of proteins using shotgun proteomics technique. Given the presence of protein homologs, one or more peptides obtained by shotgun MS/MS methods may be assigned to multiple proteins. In order to remove redundant proteins, the total proteins identified were subjected to group combination using the in-house software Buildsummary. We obtained 234 non-redundant proteins (234/648, 36.11%) in healthy plasma samples, and 272 non-redundant proteins (272/712, 38.20%) in footrot plasma samples. The lower number of the non-redundant proteins indicates that there are many redundant proteins in the current database of Bovidae proteins in NCBI. Taking into account the analysis of potential differential proteins between plasma samples from healthy and footrot-affected cattle, the high-confidence proteins among the non-redundant proteins were screened further by the standard of a unique peptide count>or = 2 or a unique peptide count = 1 but a total count>or = 4. A total of 123 high-confidence proteins were found in the healthy plasma sample, which accounted for 18.98% (123/648) of the total number of proteins and 52.56% (123/234) of the non-redundant proteins. A total of 138 high-confidence proteins were found in the footrot plasma sample, which accounted for 19.38% (138/712) of the total number of proteins and 50.74% (138/272) of the non-redundant proteins. These data demonstrate that the original proteins obtained directly using the shotgun proteomics technique comprised only less than 20% high-confidence proteins. In healthy plasma, two unknown proteins and nine predicted proteins were identified. In footrot plasma, three unknown proteins and fourteen predicted proteins were identified. These unknown and predicted proteins will enrich the bovine plasma proteomics database. The numbers of proteins identified from the footrot plasma sample, including the total number of proteins, non-redundant proteins, high-confidence proteins and potential differential proteins, were all higher than those of the healthy plasma sample.

Molecular weight and isoelectric points are two important indicators of the characteristics of a protein. Analysis of the molecular weight indicated that the high-confidence proteins of the footrot plasma sample showed significant differences from healthy plasma proteins in size ranges of 30 kDa–50 kDa, 60 kDa–70 kDa and >100 kDa. The analysis of isoelectric points (p*I*) revealed that the number of high-confidence proteins in the footrot plasma sample was significantly different from that of the healthy plasma sample in the range p*I* 4–10. The Gene Ontology (GO) database is now used widely to describe protein function in a standardized format [26]. In the GO categories of high-confidence proteins from healthy plasma, 78.05% (96/123) proteins, 73.17% (90/123) proteins, and 78.05% (96/123) proteins were annotated in cellular components, biological process and molecular functions, respectively. In the GO categories of high-confidence proteins from footrot plasma, 77.54% (107/138) proteins, 73.19% (101/138) proteins, and 76.81% (106/138) proteins were annotated in cellular components, biological processes and molecular functions, respectively. In this study, there remained a small number of the high-confidence proteins that had no assigned GO terms. This was partially due to the novel unknown or putative proteins, and also due to the limitation of the coverage of the current GO annotation system. In GO categories, these annotated high-confidence proteins from the footrot plasma sample showed significant differences from those of the healthy plasma sample in terms of cellular components, molecular functions, and biological processes. In cellular components categories, the vesicle-related proteins were found specifically in the footrot plasma sample. The vesicles are a small bubble within cell, and are thus a type of organelle. The vesicles are a basic tool used by the cell for organizing cellular substances, and perform a variety of functions, including metabolism, transport, buoyancy control, enzyme storage, and acting as chemical reaction chambers [27]. Of 16 PDE proteins in footrot plasma, the keratan sulfate proteoglycan, centromere protein F, desmoplakin and similar to superficial zone protein, involving cell adhesion and cytoskeletal proteins, exhibit a certain correlation with the vesicles in cellular components GO categories. Emergence of the vesicle-related proteins could represent a special change of the cellular components during the development phase of footrot. In footrot plasma, a total of eight differential biological processes are found to focus on the metabolic processes, regulations, transports, and establishment of localization. Of these, the regulation processes, which account for 50%, are significantly differential biological processes in the footrot plasma samples. In the footrot plasma, 11 of the 12 differential molecular functions focused on binding activity, involving in iron ions, lipoproteins, and alcohols, in which the differential iron ion binding activity caused our concerns. Emergence of the iron ion binding proteins could reflect the increase of the iron ion concentration in the footrot plasma samples to a certain extent. The changes of the iron ion concentration may have a certain relationship with erythrocyte hemolysis and live damage caused by *F. necrophorum* infection [28], [29]. Furthermore, the decreases of oxygen-carrying capacity of red blood cells because of the hemolysis would be more conducive to the anaerobic infection of *F. necrophorum* [30]. It has been proven that calcium is needed for normal claw growth and integrity, and plays an integral role in the keratinization and cornification process [31], [32]. Compared with the healthy plasma, the loss of the calcium ion binding proteins in footrot plasma samples may promote the development and progression of footrot in dairy cattle. The above-described data supported the hypothesis that the numbers, types and functions of plasma proteins had experienced great changes during the pathogenesis of footrot in the dairy cattle.

Differential proteins, or marker proteins, have become an important target of proteomics research. In this study, a total of 22 potential differentially expressed proteins were found, among which 16 proteins (2.24%) occurred in footrot plasma and 6 proteins (0.92%) in healthy plasma. The potential differential expression (PDE) proteins in each plasma sample comprised a very low percentage of the total number of proteins. Of these PDE proteins, the seven proteins in the footrot plasma, haptoglobin, SERPINA10 protein, afamin precursor, haptoglobin precursor, predicted peptidoglycan recognition protein L (PGRP-L), apolipoprotein D, and keratan sulfate proteoglycan (KS-PG), may be valuable for use as diagnostic biomarkers and in elucidation of the pathogenesis of footrot. Among the seven potential footrot-associated proteins, haptoglobin, haptoglobin precursor, and afamin precursor have been reported to be acute phase proteins, which are an integral part of the acute phase response of innate immunity [33]–[35]. The APPs have been shown to be valuable biomarkers because increases can occur with inflammation, infection, neoplasia, stress, and trauma. In past decades, haptoglobin has been shown to be a useful biomarker for monitoring the occurrence and severity of inflammatory responses in cattle with mastitis, pneumonia, enteritis, peritonitis, endocarditis, abscesses, endometritis and hoof disease [12], [13], [36], [37]. Here, haptoglobin and its precursor were verified as plasma biomarkers of footrot in dairy cattle. Among the seven potential footrot-associated proteins, haptoglobin precursor, afamin precursor, apolipoprotein-D have been reported as tumor biomarkers [38]–[40]. The identification of low-abundance serum proteins, such as tumor biomarkers, further supports the validity of the shotgun proteomics technique used in our experiment. The SERPINA10 protein, which belongs to the family of serine proteinase inhibitors, is involved in blood coagulation, complement activation, fibrinolysis, angiogenesis, inflammation, and tumor suppression [34]. It is thought that the emergence of SERPINA10 protein in plasma from footrot-affected cattle represents a defense response of the host against footrot caused by *Fusobacterum necrophorum* infection. Compared with the other five proteins, the PGRP-L and KS-PG may attract more attention as a consequence of their effects in the pathogenesis of footrot. The peptidoglycan recognition protein is required for the induction of antibacterial peptide genes in response to infection in insects and mammals [41]–[43]. The predicted PGRP-L may be necessary for recognition of the innate immune activators of the Gram-negative anaerobic bacterium *F. necrophorum*. If this is the case, the presence of the predicted PGRP-L in bovine plasma may be used as an indicator or biomarker of *F. necrophorum* infection. Keratan sulfate is any of several sulfated glycosaminoglycans that have been found especially in the cornea, cartilage, and bone. The keratan sulfate in blood has been shown to be a marker of cartilage catabolism [44], [45]. Footrot in dairy cattle, caused by *F. necrophorum* infection, is characterized by suppuration, necrosis, and corruption of the hoof tissue. If left untreated, the infection can progress into the joint space or tendon sheath, producing permanent damage. The PDE protein KS-PG, which was found in the plasma from footrot-affected dairy cattle, may reflect catabolism of hoof cartilage, and it has been suggested to be a potential marker for evaluation of foot damage in dairy cattle. In our studies, the monoclonal antibodies against human keratan sulfate and peptidoglycan recognition proteins (PGRPs) had been selected to valid the presence of two highlighted proteins KS-PG and PGRP-L in footrot and healthy plasma samples. However, only negative results were obtained in western blotting and ELISA. This data suggests that there may be some differences in both KS-PG and PGRP-L between human and dairy cattle, resulting in the antibody’s invalidity. So, further related studies are needed to confirm roles of the two highlighted proteins KS-PG and PGRP-L in footrot. To reduce the potential defect, the IgGs concentrations of the plasma samples of both healthy dairy cattle and those affected by footrot were validated by ELISA. The inceased IgGs in the footrot plasma not only represent an innate immune response of the diseased cattle to *F. necrophorum* infection, but also provide a potential support for the validity of the shotgun proteomics approach used in our study. Additionally, in healthy plasma samples, a total of six potential differential proteins were found, among which ADIPOQ protein, fibrinogen alpha chain precursor and angiotensinogen may be involved in the pathogenesis of footrot in dairy cattle [46]–[51].

To the best of our knowledge, this is the first report of plasma proteomics analysis of dairy cattle affected by footrot using the shotgun proteomics technique. Although there are still some shortcomings, the shotgun technique shows high efficiency in the identification of plasma proteins. In our current research, the protein profiles of plasma from healthy and footrot-affected dairy cattle were characterized fully using shotgun proteomics methods. We not only identified the increased IgGs, innate immune recognition molecules, acute phase proteins, regulatory proteins, and cell adhesion and cytoskeletal proteins in the footrot plasma sample, but also obtained some interesting proteins, such as predicted PGRP-L and KS-PG, which have been reported only occasionally in studies of cattle disease, especially footrot in dairy cattle. The basic information reported here will increase our understanding of bovine plasma protein profiling, and will assist in further studies of control strategies for footrot in dairy cattle.

## Supporting Information

Figure S1 **Mass spectrum base peak of plasma samples.** C1–C4. Mass spectrum base peak of the pooled healthy plasma sample, respectively; S1–S4. Mass spectrum base peak of the pooled footrot plasma sample, respectively.
(RAR)

Table S1 **The identified protein lists of plasma samples.** A. The identified protein lists of the pooled healthy plasma sample; B. The identified protein lists of the pooled footrot plasma sample.
(RAR)

Table S2 **The GO categories of the identified high-confidence proteins.** A. The GO categories of the high-confidence proteins identified from the pooled healthy plasma sample; B. The GO categories of the high-confidence proteins identified from the pooled footrot plasma sample. CC = cellular components; BP = biological processes; MF = molecular functions.
(RAR)
